# Immune-related pulmonary toxicities of checkpoint inhibitors in non-small cell lung cancer: Diagnosis, mechanism, and treatment strategies

**DOI:** 10.3389/fimmu.2023.1138483

**Published:** 2023-04-04

**Authors:** Xinyu Guo, Shi Chen, Xueyan Wang, Xiaowei Liu

**Affiliations:** Laboratory of Integrative Medicine, Clinical Research Center for Breast, State Key Laboratory of Biotherapy, West China Hospital, Sichuan University, Chengdu, Sichuan, China

**Keywords:** immune checkpoint inhibitors, immune-related adverse events (IRAE), pneumonitis, non-small cell lung cancer, treatment

## Abstract

Immune checkpoint inhibitors (ICI) therapy based on programmed cell death-1 (PD-1) and programmed cell death ligand 1 (PD-L1) has changed the treatment paradigm of advanced non-small cell lung cancer (NSCLC) and improved the survival expectancy of patients. However, it also leads to immune-related adverse events (iRAEs), which result in multiple organ damage. Among them, the most common one with the highest mortality in NSCLC patients treated with ICI is checkpoint inhibitor pneumonitis (CIP). The respiratory signs of CIP are highly coincident and overlap with those in primary lung cancer, which causes difficulties in detecting, diagnosing, managing, and treating. In clinical management, patients with serious CIP should receive immunosuppressive treatment and even discontinue immunotherapy, which impairs the clinical benefits of ICIs and potentially results in tumor recrudesce. Therefore, accurate diagnosis, detailedly dissecting the pathogenesis, and developing reasonable treatment strategies for CIP are essential to prolong patient survival and expand the application of ICI. Herein, we first summarized the diagnosis strategies of CIP in NSCLC, including the classical radiology examination and the rising serological test, pathology test, and artificial intelligence aids. Then, we dissected the potential pathogenic mechanisms of CIP, including disordered T cell subsets, the increase of autoantibodies, cross-antigens reactivity, and the potential role of other immune cells. Moreover, we explored therapeutic approaches beyond first-line steroid therapy and future direction based on targeted signaling pathways. Finally, we discussed the current impediments, future trends, and challenges in fighting ICI-related pneumonitis.

## Introduction

Recent years, with the understanding of tumor immune escape, multiple immune checkpoints have been identified for cancer immunotherapy therapy, including PD1/PD-L1, CTLA4, HLA-E/CD94-NKG2A, etc. (1–4). Non-small cell lung cancer (NSCLC) is the highest proportion of all lung cancers (80% - 85%) (5). Once diagnosed, most of them are in a locally advanced state, and the 5-year survival rate is less than 3% (6, 7). Immune checkpoint inhibitors (ICI) therapy has changed the treatment paradigm of advanced non-small cell lung cancer (NSCLC) and prolonged the 5-year overall survival rate to 23.2% (7–12). However, ICI commonly induces the disorder of immune homeostasis, which damages various normal tissues and organs, termed immune-related adverse events (iRAEs) (13, 14). About 60-80% ICI treated patients suffer iRAEs, including lung, dermatologic, gastrointestinal, renal, ophthalmic, neurologic, endocrine, musculoskeletal, hematologic, and cardiovascular toxicity (15–17). Patients who suffer severe iRAEs should immediately or even permanently discontinue ICI therapy due to the higher severity and recurrence possibility (13).

Checkpoint inhibitor pneumonitis (CIP) is one of the most severe and life-threatening iRAEs, especially in patients who suffer from NSCLC. In NSCLC patients, the tumor has destroyed the lung function, resulting in the patients receiving ICI with a higher risk of CIP. The incidence of CIP in NSCLC in real-world settings is about 7-19%, which is significantly higher than the incidence of 3-5% in other tumors, such as melanoma (6, 10, 18–27). In a retrospective study of 276 NSCLC patients treated with PD-1/PD-L1 inhibitors, the incidence of CIP is about 15.2% (24). In another study, the incidence raised to 19% in NSCLC patients receiving anti-PD-1/PD-L1 therapy (28). The typical characteristics of CIP are dyspnea, cough, hypoxia, and along with pulmonary infiltrates on chest imaging. However, accurate diagnosis and treatment of CIP in the clinic is still challenging. The radiographic images of CIP are varied and susceptible to interfere by tumors (29). Furthermore, it is difficult to distinguish CIP from infection, chemotherapy, and radiotherapy induced pneumonitis. Generally, patients with CIP are recommended to be treated with steroids. However, in serious patients, such as Common Toxicity Criteria for Adverse Events (CTCAE) grade 3 or higher, patients should discontinue ICI therapy and receive immune-suppressive treatment (30). As a result, patients with NSCLC, who escaped death from CIP, may also experience tumor recurrence. Therefore, a great deal of effort should be focused on the CIP of NSCLC.

In this review, we summarize the diagnosis, pathogenesis, and treatment strategies of CIP. Except for the multi-angle monitoring and systemic examination, we talk about the artificial intelligence (AI) which can aid in the early diagnosis of CIP. We summarize the possible pathogenic mechanisms, including disordered T cell subsets, the increase of autoantibodies, cross-antigens reactivity, and potential role of other immune cells. Moreover, we highlight the existing and future potential treatment measures for CIP, including corticosteroids, immunosuppressants, cytokine blockade, and signaling pathways inhibition. Finally, we discuss the current impediments, future trends, and challenges in fighting ICI-related pneumonitis.

## Current and emerging diagnostic strategies for CIP

**Figure 1:**
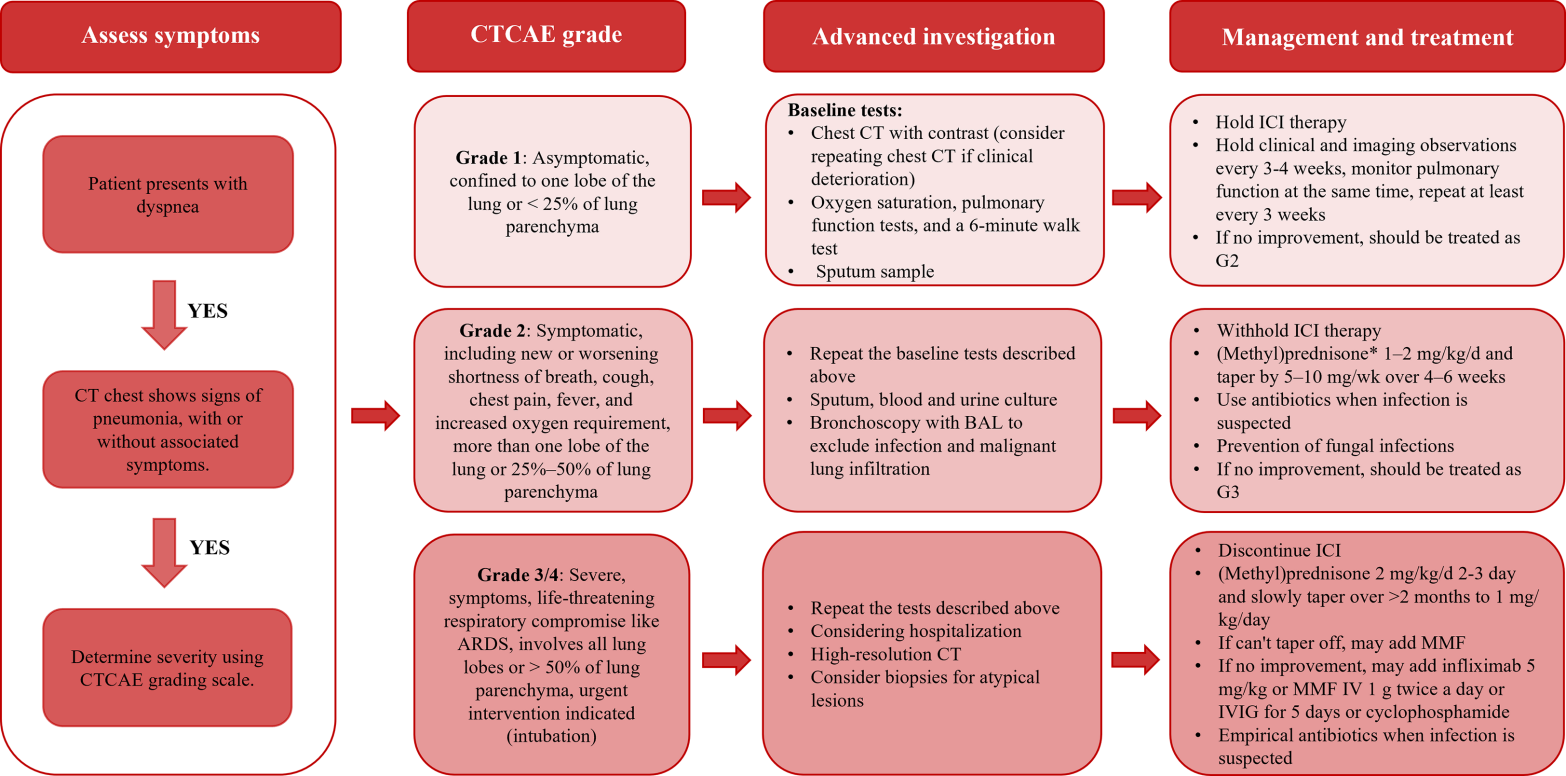
The diagnosis and management procedure of CIP in the clinic.

Commonly, once ICI-treated NSCLC patients with the characteristics of CIP, such as dyspnea, cough, and hypoxia, should be suspected and receive the standard diagnostic procedure to confirm (**Figure 1**). The radiological examination is the common strategy for diagnosing pneumonia. However, the radiology examination is difficult to distinguish CIP from common pneumonia caused by radiation, infections, and chemotherapy drugs. In recent years, a variety of serological markers, pathological markers, and AI have been developed and applied to diagnose CIP, which may change the current dilemma faced in CIP diagnosis.

### Radiology examination

Radiology examination is the routine method of diagnosing pneumonia. The imaging characteristics of CIP are nodular, reticulation, consolidation, ground-glass opacity (GGO), leaflet septal thickening, and opaque cord-like structure (31). According to the American Thoracic Society/European Respiratory Society (ATS/ERS) classification of interstitial pneumonia, the imaging characteristics of CIP mainly are nonspecific interstitial pneumonia (NSIP)-like, cryptogenic organizing pneumonia (COP)-like, hypersensitivity pneumonitis (HP)-like, and acute interstitial pneumonia (AIP)/acute respiratory distress syndrome (ARDS)-like, with COP (65%) being the most common, followed by NSIP (15%) (31, 32). Moreover, the radiographic classification of these pneumonia correlated with the clinical severity of pneumonia, with AIP/ARDS having the highest severity level, followed by COP (33). Clinically, Suresh et al. found that CIP manifested in a variety of radiographic modes, from COP to predominantly GGO or interstitial patterns (26). In this case, due to the wide range of imaging features of CIP and the lack of typicality, imaging diagnosis is difficult to distinguish CIP from infection and radiation-induced pneumonia, which will affect the accuracy of subsequent treatment.

### Serologic markers testing

Serologic markers, including cytokines and leukocytes, can be used to predict and diagnose CIP (34). For example, Lin et al. found that lung cancer patients with CIP were characterized by increased levels of IL-6, IL-10, and lactate dehydrogenase, decreased levels of albumin and absolute lymphocyte count (ALC) (35). Elevated levels of anti-CD74 autoantibodies have been found to be a potential predictor of CIP development and may be useful in identifying patients who may develop pneumonitis (36). Pavan et al. found that elevated neutrophil-to-lymphocyte ratio (NLR) and platelet-to-lymphocyte ratio (PLR) might be associated with the occurrence, severity, and subsequent prognosis of iRAEs (37). In another study, researchers found that the decrease in eosinophils was closely correlated with the CIP, especially for the high grades CIP (38). Importantly, the ratio of the percentage of eosinophils to the percentage of eosinophils at the onset of CIP is an essential marker for distinguishing CIP from pneumonia caused by bacterial infection and cancer progression (39). Taken together, serologic markers are extremely beneficial for improving the early diagnosis and clinical decision-making of CIP, and more research in this area is needed in the future.

### Pathology test

Pathology testing is not necessary for CIP but is valuable to distinguish CIP from infections radiation, and chemotherapy induced pneumonia. Naidoo et al. performed lung biopsy on 11 patients, and histopathological examination showed that CIP manifested as interstitial pneumonia, organizing pneumonia, and diffuse alveolar injury. Among them, interstitial pneumonia is found to have an increase in eosinophils and poor granuloma formation (40). However, in Imran’s study, none of the 6 CIP patients showed an increase in eosinophils, granulomatous inflammation, or necrosis (41). This variability may be due to the relative limitation of sample size, since lung tissue from NSCLC patients is often biopsied through the bronchial tube. Nevertheless, it is worth expecting that with the development of pathomics and AI, the limitations caused by the small sample size can be effectively addressed.

### Artificial intelligence fuels CIP diagnosis

In recent years, AI algorithms integrating multi-omics technologies have been widely used in cancer screening, diagnosis, and prognosis prediction, which provides new directions for future CIP diagnosis (42). Several studies have integrated imaging data, serological data, and clinical reports data with AI to diagnose and predict CIP in ICI-treated patients (43). In a retrospective study, by analyzing the CT radiomics data, Qiu et al. distinguished CIP from radiation pneumonitis in 126 advanced-stage NSCLC pneumonitis (44). Similarly, by systematically analyzing the baseline chest computed tomography images of patients with or without CIP, Colen et al. summarized the radiomics features that could distinguish and predict the risk of CIP with an accuracy of 100% (p=0.0033) (45). In another study, Park et al. proposed the likelihood of spectroscopy-based serum proteomic features for predicting the occurrence and prognosis of iRAEs, which assist in the diagnosis of CIP (46). For real clinical data, Hindocha et al. developed an informatics algorithm that integrated AI with CT reports and electronic health records to identify the CIP of ICI-treated patients and provided new real-world data on the incidence, severity and management of CIP (47). Moreover, AI algorithms can process large volumes of data from pathological sections, helping pathologists to diagnose iRAE (48). For example, by analyzing the H&E-stained colonic tissue slides, Kobayashi et al. trained a deep learning model that efficiently identified the colitis, which can be used to diagnose and classify colitis grades in ICI-treated patients (49). Although the integration of AI and pathology tests has not been applied to CIP diagnosis this can’t deny its great potential in the diagnosis of CIP. Taken together, AI algorithms will greatly improve the diagnostic efficiency and accuracy of CIP and improve clinical decision-making.

### Utility of bronchoscopy and diagnostic strategies

Bronchoscopic alveolar lavage fluid (BALF) can diagnose lung infection and interstitial pneumonia by changes in immune cells in the lavage fluid, which is not currently commonly used in the diagnosis of CIP. However, BALF is a worthwhile option when atypical infections (e.g., fungi, pneumocystis carinii pneumonia, viruses) need to be excluded and the cause of CIP needs to be investigated (30). Studies by Sabino et al. have pointed to the possibility of BALF in the diagnosis of CIP. They performed bronchoalveolar lavage (BAL) analysis on five patients with CIP, which typically showed an increase of lymphocyte and CD8^+^ T cells and a reversal of the CD4/CD8 ratio. Moreover, the grade of adverse events correlated with the degree of CD3^+^ HLA-DR^+^ T cell activation (50). This suggests that changes in immunological cells in alveolar lavage fluid can guide the clinical treatment of CIP.

Moreover, oxygen saturation, pulmonary function tests, and 6-minute walk test should be performed on any patient with suspected pneumonia to assess the specific condition of patient’s lung function, in which pulmonary function tests can be useful in monitoring the response to the treatment of patients in the management of CIP (30, 51). What’s more, the most important indicator to pay attention to is oxygen saturation, because it can directly reflect whether the body is hypoxic, which is very important for the CTCAE rating of pneumonia patients (30).

## Mechanisms of CIP

ICIs specifically block the mutual recognition of tumor cells with T cells and reactivate T cell-mediated cellular immunity to kill tumor cells (52–54). Simultaneously, these inhibitors also cause excessive activation of immunity in normal tissues to generate iRAEs (15, 55). Following is a discussion of the potential mechanisms of CIP proposed in existing studies.

### Disordered T cell subsets

The imbalance of T cell subsets, including the changes of CD8^+^ T cells and CD4^+^ T cells, has been considered involved in the occurrence of iRAEs. Recently, Suzuki et al. reported that CD8^+^ T cells significantly increased in BALF, which is closely related to the occurrence of CIP (56). The penetration of CD4^+^ T cells, represented by Th1 and Th17 cells, has also been implicated in a variety of iRAEs, including colitis, nephritis, pneumonia, and dermatological complications (57, 58). In a systematic study, Kim et al. analyzed the lymphocytes from BALF of ICI-treated patients. They found T cell clones were significantly expanded, especially for IFN-γ^+^ IL-17^-^ CD8^+^ T and CXCR3^+^ CCR6^+^ Th17/Th1 cells, suggesting the expansion of T cells plays a critical role in CIP (59). In addition, because Treg cells express CTLA-4, the anti-CTLA-4 antibody can regulate Treg cells in the tumor microenvironment and induce iRAEs by abolishing the inhibitory function of Tregs (60). Suresh et al. found that the expressions of CTLA-4 and PD-1 on BALF Tregs in CIP patients were decreased, suggesting that functional inhibition of Tregs may associated with the occurrence of CIP (61). Taken together, the increase of activated T cells and the decrease of suppressor T cells may result in CIP.

### Unbalanced inflammatory factors and autoantibodies

Unbalanced cytokines and autoantibodies secretion are other induction factors for CIP. The relationship between cytokines and iRAE was initially observed in melanoma patients who received ICI therapy. In an ICI-treated melanoma cohort, Lim et al. found that the levels of plasma cytokines, including G-CSF, GM-CSF, FRACTALKINE, FGF-2, IFNa2, IL-12p70, IL-1a, IL1, IL-1RA, IL-2, and IL-13, were associated with the development of advanced iRAEs (62). Khan et al. demonstrated that CX-C motifera chemokine ligands (CXCLs) were strongly associated with the occurrence of iRAEs. Among them, CXCL9, CXCL10, and CXCL11 bound to the C-X-C motifoligentine receptor (CXCR) 3 to activate T cells, which promotes the progression of iRAE (63). For the mechanism of CIP, multiple studies manifested that the increase of inflammatory factors, C-reactive protein (CRP), IL-6, IL-17, and IL-35, were related to the occurrence of CIP (27, 35). In a prospective study, Suresh et al. demonstrated that proinflammatory and chemotactic cytokines in BALF were significantly correlated with CIP (58). On the other hand, multiple studies have shown autoantibodies, such as rheumatoid factor (RF), antinuclear antibodies, and antithyroglobulin, resulted in patients easier to suffer from iRAE (64, 65). Tahir et al. found that the levels of anti-CD74 autoantibodies in patients with CIP increased about 1.34-fold, suggesting the increase in autoantibodies is related to CIP (36). Overall, the increased levels of various cytokines and autoantibodies may result in CIP.

### Cross-antigens reactivity

T cells are activated during antigen cross-presentation, which may be an important reason for promoting the progression of iRAEs. This mechanism has been demonstrated in a patient with fulminant myocarditis who underwent a combination of ipilimumab and nivolumab, whose tumor cells simultaneously expressed cardiomyocyte-specific antigens, suggesting a strong link between antigen cross-presentation and myocarditis (66). Another study found T cell clones were shared between skin and tumors in patients with skin-associated iRAEs, which also suggested the important role of antigen cross-presentation (67). On the other hand, tumor destruction and lysis caused by ICI treatment can also cause epitope spread (ES), leading to the destruction of normal tissue (68). ES has been reported in patients who received tumor vaccines, adoptive cell metastasis therapy, or anti-CTLA-4 therapy (69–71). Although there have been no definitive studies to prove whether CIP is associated with the cross-presentation of antigens and ES, this underlying mechanism cannot be ignored.

### Potential role of other immune cells

As essential components of humoral immunity and initial immunity, B cells and NK cells may also contribute to the development of CIP. Studies have shown that blockade of the PD-1/PD-L1 pathway promotes the activation, proliferation, and secretion of B cells (72). Similarly, Das et al. found that in patients with anti-CTLA-4 and anti-PD-1 combined therapy resulted in the levels of circulating B cells decreased and increased the levels of CD21^lo^ B cells and plasmablasts, which were strongly associated with iRAEs (73). They found that detecting the changes of B cells in blood could predict the occurrence of iRAEs. NK cells are a type of innate immune surveillance cells. Previous studies found NK cells expressed PD-1 protein and were involved in the immunosurveillance of tumors (74). When ICIs were administrated, NK cells were activated and released pro-inflammatory factors, which further promoted inflammation and damaged normal lung tissue (75). These results suggest that other immune cells also regulate the occurrence of iRAEs, and future research should be focused on this area.

## Management and new treatment strategies for CIP

If ICI-treated NSCLC patients were diagnosed with CIP, they need to be assessed for severity in accordance with CTCAE and carry out hierarchical management (**Figure 1** and **Table 1**) (30). According to the CTCAE score, CIP can be divided into four grades. For patients with grade 1 pneumonia, it is recommended to perform clinical and imaging observations every 3-4 weeks, and monitor pulmonary function at the same time, review at least every 3 weeks (76, 77, 79, 97). When grade 2 pneumonitis has developed, further treatment with high-dose corticosteroids ought to be used. If higher-grade pneumonia occurs, the ICI treatment needs to be forbidden for life, and the patient needs to be hospitalized, and the option of adding infliximab, tocilizumab, Intravenous immunoglobulin (IVIG), mycophenolate mofetil, and cyclophosphamide need to be considered when high-dose corticosteroids are not effective (78, 97).

**Table 1:** Management and treatment strategies of CIP.

Therapy	Mechanism	Drugs	Status of application	Study summary	Refs
Corticosteroid	Suppress immune cells and inflammatory cytokines	(Methyl)prednisolone	First-line option	Corticosteroid is the first-line option for the treatment of CIP recommended by guidelines, and IVIG and immunosuppressants are recommended for high-grade refractory iRAEs.	(76–80)
IVIG	Downregulate B cell function and antibody production, neutralize pathogenic autoantibodies already present in the body	Immunoglobulin	Clinical trial		
Immunosuppressant	Inhibit immune cell proliferation and function, reduce antibody immune response	Mycophenolate mofetil, Cyclophosphamide	Clinical trial		
Target cytokines	TNF-α inhibitor	Infliximab	Clinical trial	Ventilatory status improved markedly with the addition of infliximab in CIP patients who did not respond to corticosteroid therapy, but in one patient experienced transient improvement and then worsened.	(81, 82)
	IL-6 inhibitor	Tocilizumab	Clinical trial	Most CIP patients who do not respond to corticosteroid therapy have significant improvement in clinical symptoms after treatment with tocilizumab.	(83)
	IL-17 inhibitor	Secukinumab	Clinical trial	Case Reports: seukinumab is effective in treating psoriasisform dermatologic toxicity.	(84)
	IL-12 inhibitor	Ustekinumab	Clinical trial	Ustekinumab can be used in the treatment of immune-mediated refractory colitis.	(85, 86)
	IL-23 inhibitor	Ustekinumab, guselkumab	Clinical trial	Both ustekinumab and guselkumab have been shown to be effective in the treatment of moderate to severe psoriasis iRAEs.	(85, 86)
	IL-1 inhibitor	Canakinumab	Clinical trial	Canakinumab is effective in the treatment of a variety of autoimmune inflammations and has great potential for iRAEs treatment.	(87)
	IL-5 inhibitor	Mepolizumab	Clinical trial	Mepolizumab can effectively reduce eosinophil count in serum and has great potential to become a treatment option for iRAEs.	(88)
	IL-13 inhibitor	Tralokinumab	Clinical trial	IL-13 is associated with severe iRAEs, and Tralokinumab, approved for atopic dermatitis, may be the treatment of choice for severe iRAEs.	(62, 89)
	C3a inhibitor	Eculizumab, ravulizumab	Clinical trial	Eculizumab and ravulizumab are FDA approved for paroxysmal nocturnal hemoglobinuria and are potentially valuable for complement-mediated iRAEs therapy.	(90)
	GM-CSF	Sargramostim	Clinical trial	In a phase II trial, the addition of sargramostim to ICI was found to be effective in reducing gastrointestinal iRAEs and CIP and improving survival.	(91)
Target integrin	α_4_ integrins inhibitor	Vedolizumab	Clinical trial	Vedolizumab is approved for inflammatory bowel disease and has a high safety profile for long-term use, which has high potential for the treatment of iRAEs.	(92)
Signaling pathways inhibitor	JAK-associated pathway inhibitors	Ruxolitinib, baricitinib, tofacitinib, upadacitinib	Clinical trial	Tofacitinib has been reported in five cases of refractory colitis, myocarditis and arthritis, with a rapid onset of action and durable responses.	(93, 94)
		Oclacitinib	Under investigation in clinical trial NCT05305066		
	BTK-associated pathway inhibitor	Evobrutinib	Under investigation in clinical trial NCT03934502	Reductions in cytokines like IL-1β, IL-6, IL-12, and IL-17 caused by BTK inhibitiors in some primary autoimmune diseases may suggests possible mechanism to abrogate iRAEs.	(95)
		Acalabrutinib	Clinical trial		
		Tirabrutinib	Under investigation in clinical trial NCT02626026		
	MNK1/2-associated pathway inhibitor	Tomivosertib	Under investigation in clinical trials	Tomivosertib can inhibit the expression of multiple pro-inflammatory cytokines, such as IL-6, IL-17, and TNF-α, which are correlated with the development of iRAEs.	(96)
	mTOR-associated pathway inhibitor	Sirolimus	Preclinical	Studies have shown that sirolimus not only alleviates colitis by reducing T cell infiltration, but also inhibits tumor growth.	(14)

iRAEs, immune-related adverse events; IVIG, intravenous immunoglobulin; CIP, checkpoint inhibitor pneumonitis.

### Corticosteroids treatment

Currently, glucocorticoid, an anti-inflammatory drug is the first choice for the treatment of CIP in clinical (77). For grade 2 pneumonia, the NCCN guidelines recommend the use of glucocorticoids and empiric antibiotics, where a moderate dose of glucocorticoid therapy (1-2 mg/kg/d) is selected. If clinical improvement happens after monitoring gradually, the dose should be gradually reduced by 5-10 mg/week and continued for 4-6 weeks to avoid the recurrence of pneumonia, during which close observation for infection is needed. When the pneumonitis reaches grade 3 or 4, pulsatile glucocorticoid therapy is required in most cases, that is, more than 250 mg of glucocorticoid therapy for several days (7, 77). However, since there is no exact clinical trial to determine the optimal duration of glucocorticoid treatment, clinical treatment at this stage is often determined based on the patient’s response to glucocorticoids. In addition, a retrospective study demonstrated that glucocorticoid therapy may promote cancer progression and reduce overall survival, so glucocorticoids should be used with greater caution (98).

### Immunosuppressants treatment

For grade 4 CIP, guidelines indicate when glucocorticoid therapy is ineffective, other immunosuppressive options should be adopted, such as IVIG and immunosuppressant therapy (77). Immunoglobulin can downregulate B cell function and antibody production and neutralize pathogenic autoantibodies already present in the body (99). Previous studies have referred to the overall rise of autoantibodies in the serum of CIP patients, which can be effectively treated with IVIG (26). For severe iRAEs, plasmapheresis need to be considered (100). As for mycophenolate mofetil and cyclophosphamide, these two drugs are common immunosuppressants, which can inhibit immune cell proliferation and function, reduce antibody immune response, and can be late candidates for high-grade CIP therapy (77). It is important that if the patient’s symptoms improve after corticosteroid use but the later dose cannot be effectively reduced, MMF can be used as steroid sparing agent as well (79).

### Targeting secreting cytokines

Recently, several studies have demonstrated that iRAEs are associated with some specific cytokines, such as tumor necrosis factor α (TNF-α), IL-6, and IL-17. Inhibiting the production of cytokines is a promising strategy to treat iRAE, and relevant inhibitors have been approved to treat iRAE (**Table 1**). Infliximab is a monoclonal antibody of TNF-α, which can achieve anti-inflammatory effect by inactivate and degrade of TNF-α (30). In the latest updated guidance, infliximab has been recommended to treat grade 4 CIP. Data from a retrospective study demonstrated that the use of infliximab was effective in improving CIP, and the same results were confirmed in a case report (81, 82). Similarly, tocilizumab, an IL-6 inhibitor used to treat rheumatological and giant cell arteritis iRAEs, is also recommended by guidelines (30). In the study of Stroud et al., tocilizumab was used to treat glucocorticoids ineffective patients and significantly relieved CIP. They also found CIP patients with the characteristics of elevated CRP, which could decrease by tocilizumab (83).

Moreover, the therapeutic effect on CIP remains to be explored for other cytokine blockers. There have been cases of effective use of the anti-IL-17 monoclonal antibody secukinumab in the treatment of intestinal and cutaneous iRAEs, although it may promote tumor immune escape (101). In addition, with the further deepening of research on the important cytokines IL-12 and IL-23 involved in the formation of iRAEs, their important role in tumor immunity and autoimmune diseases has been valued (62, 102). The IL-12/23 inhibitor ustekinumab and the IL-23 inhibitor guselkumab have been successfully applied to the treatment of psoriasis (103). Furthermore, the IL-13 blocker tralokinumab has been approved for moderate to severe atopic dermatitis, and its use in the treatment of iRAEs remains to be explored (89). Notably, mepolizumab is an anti-IL-5 monoclonal antibody that has been shown to lower blood eosinophil counts, which may be used as an adjunct therapy for CIP (88). Although research evidence about the application of cytokines inhibitor in iRAEs is lacking, the development of targeted cytokine therapies has extraordinary potential.

### Targeting signaling pathways - the future direction

Cytokines often act as messengers by activating signaling pathways within cells, so to a certain extent, it is possible to achieve the treatment of iRAEs by targeting signaling pathways. The mTOR pathway regulates innate and adaptive immune responses and is a key factor in the regulation of T cell function, and its inhibitor sirolimus is often used to maintain immune tolerance and prevent organ transplant rejection (104, 105). In our previous study, we found that sirolimus not only inhibited tumor growth but also prevented colitis by inhibiting the infiltration of T cells, suggesting its great potential for the treatment of iRAEs and tumors (14, 106). The JAK–STAT pathway is induced by a number of closely related cytokines, such as IL-6, IL-12, IL-23, and IL-17, which are essential for the immune mechanisms of autoimmune diseases and cancer progression. In addition, JAK-STAT pathway is also an important pathway for IFN regulation of innate and adaptive immunity, and abnormal IFN signaling has been shown to lead to autoimmune diseases. Five JAK-STAT inhibitors (ruxolitinib, baritinib, tofacitinib, oclacitinib, and upadacitinib) have been approved for autoimmune diseases. Two patients with iRAEs-associated myocarditis and one patient with iRAEs-associated arthritis have been reported to have significant remission with tofacitinib, with rapid onset of action and long-lasting response (107, 108). Evidently, the development of cell signaling pathway-oriented therapeutic strategies is worth looking forward to.

## Conclusion and prospects

At present, both the diagnosis and treatment of CIP need to be solved urgently in clinical. The diagnosis of CIP is an exclusionary diagnosis, and the uncertainty of various diagnostic indicators will greatly delay the diagnosis and subsequent treatment. Although imaging techniques, pulmonary function tests, pathology tests, and serological tests have been applied in CIP diagnosis (34). It is still urgent to develop new tools with high accuracy for early diagnosis of CIP. Another critical aspect is the need to focus more on predicting high-risk CIP for identifying high-risk patients and subsequent close observation (109). From a treatment perspective, the timing of ICI treatment discontinuation and initiation of glucocorticoid therapy, the dosage, usage, and duration of glucocorticoid therapy need to be investigated (78). In addition, the existing treatment methods will affect the prognosis of NSCLC to a certain extent, and whether it can develop effective treatment of side effects without affecting the process of primary NSCLC is the key to the current treatment strategy research. Given this situation, the choice of cytokine antagonists or blockers of signaling pathways may open a new door for the treatment of CIP, which requires extensive research to demonstrate.

## Author contributions

All authors listed have made a substantial, direct, and intellectual contribution to the work and approved it for publication.

## References

[1] Hodi FS O'Day SJ McDermott DF Weber RW Sosman JA Haanen JB. Improved survival with ipilimumab in patients with metastatic melanoma. New Engl J Med (2010) 363(8):711–23. doi: 10.1056/NEJMoa1003466 PMC3549297 20525992

[2] Schachter J Ribas A Long GV Arance A Grob J-J Mortier L. Pembrolizumab versus ipilimumab for advanced melanoma: Final overall survival results of a multicentre, randomised, open-label phase 3 study (Keynote-006). Lancet (2017) 390(10105):1853–62. doi: 10.1016/s0140-6736(17)31601-x 28822576

[3] Liu X Song J Zhang H Liu X Zuo F Zhao Y. Immune checkpoint hla-E:Cd94-Nkg2a mediates evasion of circulating tumor cells from nk cell surveillance. Cancer Cell (2023) 41(2):272–87.e9. doi: 10.1016/j.ccell.2023.01.001 36706761

[4] Zhao Y Liu X Liu X Yu J Bai X Wu X. Combination of phototherapy with immune checkpoint blockade: Theory and practice in cancer. Front Immunol (2022) 13:955920. doi: 10.3389/fimmu.2022.955920 36119019 PMC9478587

[5] Madden K Kasler MK. Immune checkpoint inhibitors in lung cancer and melanoma. Semin Oncol Nurs (2019) 35(5):150932. doi: 10.1016/j.soncn.2019.08.011 31561846

[6] Zhang Q Tang L Zhou Y He W Li W. Immune checkpoint inhibitor-associated pneumonitis in non-small cell lung cancer: Current understanding in characteristics, diagnosis, and management. Front Immunol (2021) 12:663986. doi: 10.3389/fimmu.2021.663986 34122422 PMC8195248

[7] Brahmer JR Govindan R Anders RA Antonia SJ Sagorsky S Davies MJ. The society for immunotherapy of cancer consensus statement on immunotherapy for the treatment of non-small cell lung cancer (Nsclc). J Immunother Cancer (2018) 6(1):75. doi: 10.1186/s40425-018-0382-2 30012210 PMC6048854

[8] Baxi S Yang A Gennarelli RL Khan N Wang Z Boyce L. Immune-related adverse events for anti-Pd-1 and anti-Pd-L1 drugs: Systematic review and meta-analysis. BMJ (2018) 360:k793. doi: 10.1136/bmj.k793 29540345 PMC5851471

[9] Zhou X Yao Z Bai H Duan J Wang Z Wang X. Treatment-related adverse events of pd-1 and pd-L1 inhibitor-based combination therapies in clinical trials: A systematic review and meta-analysis. Lancet Oncol (2021) 22(9):1265–74. doi: 10.1016/s1470-2045(21)00333-8 34391508

[10] Zhai X Zhang J Tian Y Li J Jing W Guo H. The mechanism and risk factors for immune checkpoint inhibitor pneumonitis in non-small cell lung cancer patients. Cancer Biol Med (2020) 17(3):599–611. doi: 10.20892/j.issn.2095-3941.2020.0102 32944393 PMC7476083

[11] Cao R Ma JT Zhang SL Sun L Liu Y Zhang XY. Rational application of the first-line chemotherapy and immune checkpoint inhibitors in advanced nonsmall cell lung cancer: A meta-analysis. Cancer Med (2019) 8(11):5033–46. doi: 10.1002/cam4.2407 PMC6718602 31297962

[12] Insa A Martin-Martorell P Di Liello R Fasano M Martini G Napolitano S. Which treatment after first line therapy in nsclc patients without genetic alterations in the era of immunotherapy? Crit Rev Oncol Hematol (2022) 169:103538. doi: 10.1016/j.critrevonc.2021.103538 34801700

[13] Jing Y Zhang Y Wang J Li K Chen X Heng J. Association between sex and immune-related adverse events during immune checkpoint inhibitor therapy. J Natl Cancer Inst (2021) 113(10):1396–404. doi: 10.1093/jnci/djab035 33705549

[14] Bai X Wang X Ma G Song J Liu X Wu X. Improvement of pd-1 blockade efficacy and elimination of immune-related gastrointestinal adverse effect by mtor inhibitor. Front Immunol (2021) 12:793831. doi: 10.3389/fimmu.2021.793831 34987517 PMC8721049

[15] Okiyama N Tanaka R. Immune-related adverse events in various organs caused by immune checkpoint inhibitors. Allergol Int (2022) 71(2):169–78. doi: 10.1016/j.alit.2022.01.001 35101349

[16] Bertrand A Kostine M Barnetche T Truchetet ME Schaeverbeke T. Immune related adverse events associated with anti-Ctla-4 antibodies: Systematic review and meta-analysis. BMC Med (2015) 13:211. doi: 10.1186/s12916-015-0455-8 26337719 PMC4559965

[17] Chuzi S Tavora F Cruz M Costa R Chae YK Carneiro BA. Clinical features, diagnostic challenges, and management strategies in checkpoint inhibitor-related pneumonitis. Cancer Manag Res (2017) 9:207–13. doi: 10.2147/CMAR.S136818 PMC5476791 28652812

[18] Reuss JE Suresh K Naidoo J. Checkpoint inhibitor pneumonitis: Mechanisms, characteristics, management strategies, and beyond. Curr Oncol Rep (2020) 22(6):56. doi: 10.1007/s11912-020-00920-z 32415399

[19] Suresh K Naidoo J Lin CT Danoff S. Immune checkpoint immunotherapy for non-small cell lung cancer: Benefits and pulmonary toxicities. Chest (2018) 154(6):1416–23. doi: 10.1016/j.chest.2018.08.1048 PMC6335259 30189190

[20] Khunger M Rakshit S Pasupuleti V Hernandez AV Mazzone P Stevenson J. Incidence of pneumonitis with use of programmed death 1 and programmed death-ligand 1 inhibitors in non-small cell lung cancer: A systematic review and meta-analysis of trials. Chest (2017) 152(2):271–81. doi: 10.1016/j.chest.2017.04.177 28499515

[21] Nishino M Giobbie-Hurder A Hatabu H Ramaiya NH Hodi FS. Incidence of programmed cell death 1 inhibitor-related pneumonitis in patients with advanced cancer: A systematic review and meta-analysis. JAMA Oncol (2016) 2(12):1607–16. doi: 10.1001/jamaoncol.2016.2453 27540850

[22] Hellmann MD Rizvi NA Goldman JW Gettinger SN Borghaei H Brahmer JR. Nivolumab plus ipilimumab as first-line treatment for advanced non-Small-Cell lung cancer (Checkmate 012): Results of an open-label, phase 1, multicohort study. Lancet Oncol (2017) 18(1):31–41. doi: 10.1016/S1470-2045(16)30624-6 27932067 PMC5476941

[23] Andruska N Mahapatra L Hebbard C Patel P Paul V. Severe pneumonitis refractory to steroids following anti-Pd-1 immunotherapy. BMJ Case Rep (2018) 2018:bcr-2018-225937. doi: 10.1136/bcr-2018-225937 PMC6194409 30301729

[24] Cui P Huang D Wu Z Tao H Zhang S Ma J. Association of immune-related pneumonitis with the efficacy of pd-1/Pd-L1 inhibitors in non-small cell lung cancer. Ther Adv Med Oncol (2020) 12:1758835920922033. doi: 10.1177/1758835920922033 32426052 PMC7222233

[25] Oshima Y Tanimoto T Yuji K Tojo A. Egfr-Tki-Associated interstitial pneumonitis in nivolumab-treated patients with non-small cell lung cancer. JAMA Oncol (2018) 4(8):1112–5. doi: 10.1001/jamaoncol.2017.4526 PMC5885195 29327061

[26] Suresh K Voong KR Shankar B Forde PM Ettinger DS Marrone KA. Pneumonitis in non-small cell lung cancer patients receiving immune checkpoint immunotherapy: Incidence and risk factors. J Thorac Oncol (2018) 13(12):1930–9. doi: 10.1016/j.jtho.2018.08.2035 30267842

[27] Wang YN Lou DF Li DY Jiang W Dong JY Gao W. Elevated levels of il-17a and il-35 in plasma and bronchoalveolar lavage fluid are associated with checkpoint inhibitor pneumonitis in patients with non-small cell lung cancer. Oncol Lett (2020) 20(1):611–22. doi: 10.3892/ol.2020.11618 PMC7285943 32565986

[28] Voong KR Hazell SZ Fu W Hu C Lin CT Ding K. Relationship between prior radiotherapy and checkpoint-inhibitor pneumonitis in patients with advanced non-Small-Cell lung cancer. Clin Lung Cancer (2019) 20(4):e470–e9. doi: 10.1016/j.cllc.2019.02.018 PMC8767572 31031204

[29] Yin J Wu Y Yang X Gan L Xue J. Checkpoint inhibitor pneumonitis induced by anti-Pd-1/Pd-L1 therapy in non-Small-Cell lung cancer: Occurrence and mechanism. Front Immunol (2022) 13:830631. doi: 10.3389/fimmu.2022.830631 35464480 PMC9021596

[30] Haanen J Obeid M Spain L Carbonnel F Wang Y Robert C. Management of toxicities from immunotherapy: Esmo clinical practice guideline for diagnosis, treatment and follow-up. Ann Oncol (2022) 33(12):1217–38. doi: 10.1016/j.annonc.2022.10.001 36270461

[31] Travis WD Costabel U Hansell DM King TE Jr. Lynch DA Nicholson AG. An official American thoracic Society/European respiratory society statement: Update of the international multidisciplinary classification of the idiopathic interstitial pneumonias. Am J Respir Crit Care Med (2013) 188(6):733–48. doi: 10.1164/rccm.201308-1483ST PMC5803655 24032382

[32] Nishino M Ramaiya NH Awad MM Sholl LM Maattala JA Taibi M. Pd-1 inhibitor-related pneumonitis in advanced cancer patients: Radiographic patterns and clinical course. Clin Cancer Res (2016) 22(24):6051–60. doi: 10.1158/1078-0432.Ccr-16-1320 PMC5161686 27535979

[33] Castanon E. Anti-Pd1-Induced pneumonitis: Capturing the hidden enemy. Clin Cancer Res (2016) 22(24):5956–8. doi: 10.1158/1078-0432.Ccr-16-2033 27683178

[34] Jia XH Geng LY Jiang PP Xu H Nan KJ Yao Y. The biomarkers related to immune related adverse events caused by immune checkpoint inhibitors. J Exp Clin Cancer Res (2020) 39(1):284. doi: 10.1186/s13046-020-01749-x 33317597 PMC7734811

[35] Lin X Deng H Yang Y Wu J Qiu G Li S. Peripheral blood biomarkers for early diagnosis, severity, and prognosis of checkpoint inhibitor-related pneumonitis in patients with lung cancer. Front Oncol (2021) 11:698832. doi: 10.3389/fonc.2021.698832 34327140 PMC8313853

[36] Tahir SA Gao J Miura Y Blando J Tidwell RSS Zhao H. Autoimmune antibodies correlate with immune checkpoint therapy-induced toxicities. Proc Natl Acad Sci U.S.A. (2019) 116(44):22246–51. doi: 10.1073/pnas.1908079116 PMC6825284 31611368

[37] Pavan A Calvetti L Dal Maso A Attili I Del Bianco P Pasello G. Peripheral blood markers identify risk of immune-related toxicity in advanced non-small cell lung cancer treated with immune-checkpoint inhibitors. Oncologist (2019) 24(8):1128–36. doi: 10.1634/theoncologist.2018-0563 PMC6693718 31015312

[38] Li Y Jia X Du Y Mao Z Zhang Y Shen Y. Eosinophil as a biomarker for diagnosis, prediction, and prognosis evaluation of severe checkpoint inhibitor pneumonitis. Front Oncol (2022) 12:827199. doi: 10.3389/fonc.2022.827199 36033529 PMC9413068

[39] Eng SS DeFelice ML. The role and immunobiology of eosinophils in the respiratory system: A comprehensive review. Clin Rev Allergy Immunol (2016) 50(2):140–58. doi: 10.1007/s12016-015-8526-3 26797962

[40] Naidoo J Wang X Woo KM Iyriboz T Halpenny D Cunningham J. Pneumonitis in patients treated with anti-programmed death-1/Programmed death ligand 1 therapy. J Clin Oncol (2017) 35(7):709–17. doi: 10.1200/jco.2016.68.2005 PMC5559901 27646942

[41] Imran S Golden A Feinstein M Plodkowski A Bodd F Rekhtman N. Immune check-point inhibitor-related pneumonitis: Acute lung injury with rapid progression and organising pneumonia with less severe clinical disease. Histopathology (2022) 81(6):724-31. doi: 10.1111/his.14704 PMC10428658 35775853

[42] Harmon SA Sanford TH Xu S Turkbey EB Roth H Xu Z. Artificial intelligence for the detection of covid-19 pneumonia on chest ct using multinational datasets. Nat Commun (2020) 11(1):4080. doi: 10.1038/s41467-020-17971-2 32796848 PMC7429815

[43] He X Liu X Zuo F Shi H Jing J. Artificial intelligence-based multi-omics analysis fuels cancer precision medicine. Semin Cancer Biol (2023) 88:187–200. doi: 10.1016/j.semcancer.2022.12.009 36596352

[44] Qiu Q Xing L Wang Y Feng A Wen Q. Development and validation of a radiomics nomogram using computed tomography for differentiating immune checkpoint inhibitor-related pneumonitis from radiation pneumonitis for patients with non-small cell lung cancer. Front Immunol (2022) 13:870842. doi: 10.3389/fimmu.2022.870842 35558076 PMC9088878

[45] Colen RR Fujii T Bilen MA Kotrotsou A Abrol S Hess KR. Radiomics to predict immunotherapy-induced pneumonitis: Proof of concept. Invest New Drugs (2018) 36(4):601–7. doi: 10.1007/s10637-017-0524-2 PMC5924418 29075985

[46] Park Y Kim MJ Choi Y Kim NH Kim L Hong SPD. Role of mass spectrometry-based serum proteomics signatures in predicting clinical outcomes and toxicity in patients with cancer treated with immunotherapy. J Immunother Cancer (2022) 10(3):e003566. doi: 10.1136/jitc-2021-003566 PMC8961104 35347071

[47] Hindocha S Campbell D Ahmed M Giorgakoudi K Sharma B Yousaf N. Immune checkpoint inhibitor and radiotherapy-related pneumonitis: An informatics approach to determine real-world incidence, severity, management, and resource implications. Front Med (Lausanne) (2021) 8:764563. doi: 10.3389/fmed.2021.764563 34790682 PMC8591134

[48] Gurcan MN Boucheron LE Can A Madabhushi A Rajpoot NM Yener B. Histopathological image analysis: A review. IEEE Rev BioMed Eng (2009) 2:147–71. doi: 10.1109/rbme.2009.2034865 PMC2910932 20671804

[49] Kobayashi S Shieh J Ruiz de Sabando A Kim J Liu Y Zee SY. Deep learning-based approach to the characterization and quantification of histopathology in mouse models of colitis. PloS One (2022) 17(8):e0268954. doi: 10.1371/journal.pone.0268954 36037173 PMC9423669

[50] Strippoli S Fucci L Negri A Putignano D Cisternino ML Napoli G. Cellular analysis of bronchoalveolar lavage fluid to narrow differential diagnosis of checkpoint inhibitor-related pneumonitis in metastatic melanoma. J Transl Med (2020) 18(1):473. doi: 10.1186/s12967-020-02650-z 33302981 PMC7727780

[51] O'Kane GM Labbé C Doherty MK Young K Albaba H Leighl NB. Monitoring and management of immune-related adverse events associated with programmed cell death protein-1 axis inhibitors in lung cancer. Oncologist (2017) 22(1):70–80. doi: 10.1634/theoncologist.2016-0164 27534573 PMC5313273

[52] Ghahremanloo A Soltani A Modaresi SMS Hashemy SI. Recent advances in the clinical development of immune checkpoint blockade therapy. Cell Oncol (Dordr) (2019) 42(5):609–26. doi: 10.1007/s13402-019-00456-w PMC12994343 31201647

[53] Kumar S Sarthi P Mani I Ashraf MU Kang MH Kumar V. Epitranscriptomic approach: To improve the efficacy of icb therapy by Co-targeting intracellular checkpoint cish. Cells (2021) 10(9):2250. doi: 10.3390/cells10092250 PMC8466810 34571899

[54] Morad G Helmink BA Sharma P Wargo JA. Hallmarks of response, resistance, and toxicity to immune checkpoint blockade. Cell (2022) 185(3):576. doi: 10.1016/j.cell.2022.01.008 35120665

[55] Postow MA Sidlow R Hellmann MD. Immune-related adverse events associated with immune checkpoint blockade. New Engl J Med (2018) 378(2):158–68. doi: 10.1056/NEJMra1703481 29320654

[56] Suzuki K Yanagihara T Matsumoto K Kusaba H Yamauchi T Ikematsu Y. Immune-checkpoint profiles for T cells in bronchoalveolar lavage fluid of patients with immune-checkpoint inhibitor-related interstitial lung disease. Int Immunol (2020) 32(8):547–57. doi: 10.1093/intimm/dxaa022 32253426

[57] Ibraheim H Perucha E Powell N. Pathology of immune-mediated tissue lesions following treatment with immune checkpoint inhibitors. Rheumatol (Oxford) (2019) 58(Suppl 7):vii17–28. doi: 10.1093/rheumatology/kez465 PMC6900915 31816081

[58] Suresh K Naidoo J Zhong Q Xiong Y Mammen J de Flores MV. The alveolar immune cell landscape is dysregulated in checkpoint inhibitor pneumonitis. J Clin Invest (2019) 129(10):4305–15. doi: 10.1172/jci128654 PMC6763233 31310589

[59] Kim ST Sheshadri A Shannon V Kontoyiannis DP Kantarjian H Garcia-Manero G. Distinct immunophenotypes of T cells in bronchoalveolar lavage fluid from leukemia patients with immune checkpoint inhibitors-related pulmonary complications. Front Immunol (2020) 11:590494. doi: 10.3389/fimmu.2020.590494 33552049 PMC7859512

[60] Zappasodi R Serganova I Cohen IJ Maeda M Shindo M Senbabaoglu Y. Ctla-4 blockade drives loss of T(Reg) stability in glycolysis-low tumours. Nature (2021) 591(7851):652–8. doi: 10.1038/s41586-021-03326-4 PMC8057670 33588426

[61] Knochelmann HM Dwyer CJ Bailey SR Amaya SM Elston DM Mazza-McCrann JM. When worlds collide: Th17 and treg cells in cancer and autoimmunity. Cell Mol Immunol (2018) 15(5):458–69. doi: 10.1038/s41423-018-0004-4 PMC6068176 29563615

[62] Lim SY Lee JH Gide TN Menzies AM Guminski A Carlino MS. Circulating cytokines predict immune-related toxicity in melanoma patients receiving anti-Pd-1-Based immunotherapy. Clin Cancer Res (2019) 25(5):1557–63. doi: 10.1158/1078-0432.Ccr-18-2795 30409824

[63] Khan S Khan SA Luo X Fattah FJ Saltarski J Gloria-McCutchen Y. Immune dysregulation in cancer patients developing immune-related adverse events. Br J Cancer (2019) 120(1):63–8. doi: 10.1038/s41416-018-0155-1 PMC6325132 30377338

[64] Kurimoto C Inaba H Ariyasu H Iwakura H Ueda Y Uraki S. Predictive and sensitive biomarkers for thyroid dysfunctions during treatment with immune-checkpoint inhibitors. Cancer Sci (2020) 111(5):1468–77. doi: 10.1111/cas.14363 PMC7226278 32086984

[65] Ghosh N Postow M Zhu C Jannat-Khah D Li QZ Vitone G. Lower baseline autoantibody levels are associated with immune-related adverse events from immune checkpoint inhibition. J Immunother Cancer (2022) 10(1):e004008. doi: 10.1136/jitc-2021-004008 PMC8804686 35091456

[66] June CH Warshauer JT Bluestone JA. Is autoimmunity the achilles' heel of cancer immunotherapy? Nat Med (2017) 23(5):540–7. doi: 10.1038/nm.4321 28475571

[67] Berner F Bomze D Diem S Ali OH Fässler M Ring S. Association of checkpoint inhibitor-induced toxic effects with shared cancer and tissue antigens in non-small cell lung cancer. JAMA Oncol (2019) 5(7):1043–7. doi: 10.1001/jamaoncol.2019.0402 PMC6487908 31021392

[68] Brossart P. The role of antigen spreading in the efficacy of immunotherapies. Clin Cancer Res (2020) 26(17):4442–7. doi: 10.1158/1078-0432.Ccr-20-0305 32357962

[69] Corbière V Chapiro J Stroobant V Ma W Lurquin C Lethé B. Antigen spreading contributes to mage vaccination-induced regression of melanoma metastases. Cancer Res (2011) 71(4):1253–62. doi: 10.1158/0008-5472.Can-10-2693 21216894

[70] Beatty GL Haas AR Maus MV Torigian DA Soulen MC Plesa G. Mesothelin-specific chimeric antigen receptor mrna-engineered T cells induce anti-tumor activity in solid malignancies. Cancer Immunol Res (2014) 2(2):112–20. doi: 10.1158/2326-6066.Cir-13-0170 PMC3932715 24579088

[71] Ramos PS Shedlock AM Langefeld CD. Genetics of autoimmune diseases: Insights from population genetics. J Hum Genet (2015) 60(11):657–64. doi: 10.1038/jhg.2015.94 PMC4660050 26223182

[72] Thibult ML Mamessier E Gertner-Dardenne J Pastor S Just-Landi S Xerri L. Pd-1 is a novel regulator of human b-cell activation. Int Immunol (2013) 25(2):129–37. doi: 10.1093/intimm/dxs098 23087177

[73] Das R Bar N Ferreira M Newman AM Zhang L Bailur JK. Early b cell changes predict autoimmunity following combination immune checkpoint blockade. J Clin Invest (2018) 128(2):715–20. doi: 10.1172/jci96798 PMC5785243 29309048

[74] Sivori S Pende D Quatrini L Pietra G Della Chiesa M Vacca P. Nk cells and ilcs in tumor immunotherapy. Mol Aspects Med (2021) 80:100870. doi: 10.1016/j.mam.2020.100870 32800530

[75] Hsu J Hodgins JJ Marathe M Nicolai CJ Bourgeois-Daigneault MC Trevino TN. Contribution of nk cells to immunotherapy mediated by pd-1/Pd-L1 blockade. J Clin Invest (2018) 128(10):4654–68. doi: 10.1172/jci99317 PMC6159991 30198904

[76] Thompson JA Schneider BJ Brahmer J Andrews S Armand P Bhatia S. Nccn guidelines insights: Management of immunotherapy-related toxicities, version 1.2020. J Natl Compr Canc Netw (2020) 18(3):230–41. doi: 10.6004/jnccn.2020.0012 32135517

[77] Brahmer JR Lacchetti C Schneider BJ Atkins MB Brassil KJ Caterino JM. Management of immune-related adverse events in patients treated with immune checkpoint inhibitor therapy: American society of clinical oncology clinical practice guideline. J Clin Oncol (2018) 36(17):1714–68. doi: 10.1200/jco.2017.77.6385 PMC6481621 29442540

[78] Rashdan S Minna JD Gerber DE. Diagnosis and management of pulmonary toxicity associated with cancer immunotherapy. Lancet Respir Med (2018) 6(6):472–8. doi: 10.1016/s2213-2600(18)30172-3 PMC7341891 29856320

[79] Puzanov I Diab A Abdallah K Bingham CO 3rd Brogdon C Dadu R. Managing toxicities associated with immune checkpoint inhibitors: Consensus recommendations from the society for immunotherapy of cancer (Sitc) toxicity management working group. J Immunother Cancer (2017) 5(1):95. doi: 10.1186/s40425-017-0300-z 29162153 PMC5697162

[80] Petri CR Patell R Batalini F Rangachari D Hallowell RW. Severe pulmonary toxicity from immune checkpoint inhibitor treated successfully with intravenous immunoglobulin: Case report and review of the literature. Respir Med Case Rep (2019) 27:100834. doi: 10.1016/j.rmcr.2019.100834 31008047 PMC6456450

[81] Cooksley T Marshall W Gupta A. Early infliximab in life-threatening immune-mediated pneumonitis. Qjm (2019) 112(12):929–30. doi: 10.1093/qjmed/hcz224 31504921

[82] Sawai Y Katsuya Y Shinozaki-Ushiku A Iwasaki A Fukayama M Watanabe K. Rapid temporal improvement of pembrolizumab-induced pneumonitis using the anti-Tnf-A antibody infliximab. Drug Discovery Ther (2019) 13(3):164–7. doi: 10.5582/ddt.2019.01032 31257354

[83] Stroud CR Hegde A Cherry C Naqash AR Sharma N Addepalli S. Tocilizumab for the management of immune mediated adverse events secondary to pd-1 blockade. J Oncol Pharm Pract (2019) 25(3):551–7. doi: 10.1177/1078155217745144 29207939

[84] Johnson D Patel AB Uemura MI Trinh VA Jackson N Zobniw CM. Il17a blockade successfully treated psoriasiform dermatologic toxicity from immunotherapy. Cancer Immunol Res (2019) 7(6):860–5. doi: 10.1158/2326-6066.CIR-18-0682 30996018

[85] Phillips GS Wu J Hellmann MD Postow MA Rizvi NA Freites-Martinez A. Treatment outcomes of immune-related cutaneous adverse events. J Clin Oncol (2019) 37(30):2746-58. doi: 10.1200/JCO.1802141. al Pe. PMC7001790 31216228

[86] Anusha S MDea. T. Ustekinumab for refractory colitis associated with immune checkpoint inhibitors. New Engl J Med (2021) 384 (6):581-3. doi: 10.1056/NEJMc2031717 33567198

[87] Dinarello CA Simon A van der Meer JW. Treating inflammation by blocking interleukin-1 in a broad spectrum of diseases. Nat Rev Drug Discovery (2012) 11(8):633–52. doi: 10.1038/nrd3800 PMC3644509 22850787

[88] Wechsler ME Akuthota P Jayne D Khoury P Klion A Langford CA. Mepolizumab or placebo for eosinophilic granulomatosis with polyangiitis. N Engl J Med (2017) 376(20):1921–32. doi: 10.1056/NEJMoa1702079 PMC5548295 28514601

[89] Duggan S. Tralokinumab: First approval. Drugs (2021) 81(14):1657–63. doi: 10.1007/s40265-021-01583-1 PMC8519819 34406631

[90] Frieri C Peffault de Latour R Sicre De Fontbrune F. Emerging drugs for the treatment of paroxysmal nocturnal hemoglobinuria. Expert Opin Emerg Drugs (2022) 27(1):33–43. doi: 10.1080/14728214.2022.2031973 35078384

[91] Hodi FS Lee S McDermott DF Rao UN Butterfield LH Tarhini AA. Ipilimumab plus sargramostim vs ipilimumab alone for treatment of metastatic melanoma. Jama (2014) 312(17):1744-53. doi: 10.1001/jama.2014.13943 PMC4336189 25369488

[92] Loftus EV Jr. Feagan BG Panaccione R Colombel JF Sandborn WJ Sands BE. Long-term safety of vedolizumab for inflammatory bowel disease. Aliment Pharmacol Ther (2020) 52(8):1353–65. doi: 10.1111/apt.16060 PMC7540482 32876349

[93] Goyal P Choi JJ Pinheiro LC Schenck EJ Chen R Jabri A. Clinical characteristics of covid-19 in new York city. N Engl J Med (2020) 382(24):2372–4. doi: 10.1056/NEJMc2010419 PMC7182018 32302078

[94] Bishu S Melia J Sharfman W Lao CD Fecher LA Higgins PDR. Efficacy and outcome of tofacitinib in immune checkpoint inhibitor colitis. Gastroenterology (2021) 160(3):932–4.e3. doi: 10.1053/j.gastro.2020.10.029 33096100 PMC11165592

[95] Haselmayer P Camps M Liu-Bujalski L Nguyen N Morandi F Head J. Efficacy and pharmacodynamic modeling of the btk inhibitor evobrutinib in autoimmune disease models. J Immunol (2019) 202(10):2888–906. doi: 10.4049/jimmunol.1800583 PMC6500888 30988116

[96] Joshi S Platanias LC. Mnk kinase pathway: Cellular functions and biological outcomes. World J Biol Chem (2014) 5(3):321–33. doi: 10.4331/wjbc.v5.i3.321 PMC4160526 25225600

[97] Haanen J Carbonnel F Robert C Kerr KM Peters S Larkin J. Management of toxicities from immunotherapy: Esmo clinical practice guidelines for diagnosis, treatment and follow-up. Ann Oncol (2017) 28(suppl_4):iv119–iv42. doi: 10.1093/annonc/mdx225 28881921

[98] Bruera S Suarez-Almazor ME. The effects of glucocorticoids and immunosuppressants on cancer outcomes in checkpoint inhibitor therapy. Front Oncol (2022) 12:928390. doi: 10.3389/fonc.2022.928390 36081549 PMC9445222

[99] Kazatchkine MD Kaveri SV. Immunomodulation of autoimmune and inflammatory diseases with intravenous immune globulin. N Engl J Med (2001) 345(10):747–55. doi: 10.1056/NEJMra993360 11547745

[100] Esfahani K Buhlaiga N Thébault P Lapointe R Johnson NA Miller WH Jr. Alemtuzumab for immune-related myocarditis due to pd-1 therapy. N Engl J Med (2019) 380(24):2375–6. doi: 10.1056/NEJMc1903064 31189042

[101] Esfahani K Miller WH Jr. Reversal of autoimmune toxicity and loss of tumor response by interleukin-17 blockade. N Engl J Med (2017) 376(20):1989–91. doi: 10.1056/NEJMc1703047 28514612

[102] Teng MW Bowman EP McElwee JJ Smyth MJ Casanova JL Cooper AM. Il-12 and il-23 cytokines: From discovery to targeted therapies for immune-mediated inflammatory diseases. Nat Med (2015) 21(7):719–29. doi: 10.1038/nm.3895 26121196

[103] Kerschbaumer A Smolen JS Dougados M de Wit M Primdahl J McInnes I. Pharmacological treatment of psoriatic arthritis: A systematic literature research for the 2019 update of the eular recommendations for the management of psoriatic arthritis. Ann Rheum Dis (2020) 79(6):778–86. doi: 10.1136/annrheumdis-2020-217163 32381564

[104] Saravia J Raynor JL Chapman NM Lim SA Chi H. Signaling networks in immunometabolism. Cell Res (2020) 30(4):328–42. doi: 10.1038/s41422-020-0301-1 PMC7118125 32203134

[105] Abdel-Wahab N Safa H Abudayyeh A Johnson DH Trinh VA Zobniw CM. Checkpoint inhibitor therapy for cancer in solid organ transplantation recipients: An institutional experience and a systematic review of the literature. J Immunother Cancer (2019) 7(1):106. doi: 10.1186/s40425-019-0585-1 30992053 PMC6469201

[106] Henderson Berg MH Del Rincon SV Miller WH. Potential therapies for immune-related adverse events associated with immune checkpoint inhibition: From monoclonal antibodies to kinase inhibition. J Immunother Cancer (2022) 10(1):e003551. doi: 10.1136/jitc-2021-003551 PMC8796266 35086945

[107] Liu Y Jiang L. Tofacitinib for treatment in immune-mediated myocarditis: The first reported cases. J Oncol Pharm Pract (2020), 1078155220947141. doi: 10.1177/1078155220947141 32781887

[108] Murray K Floudas A Murray C Fabre A Crown J Fearon U. First use of tofacitinib to treat an immune checkpoint inhibitor-induced arthritis. BMJ Case Rep (2021) 14(2):e238851. doi: 10.1136/bcr-2020-238851 PMC7868229 33541985

[109] Naidoo J Nishino M Patel SP Shankar B Rekhtman N Illei P. Immune-related pneumonitis after chemoradiotherapy and subsequent immune checkpoint blockade in unresectable stage iii non-Small-Cell lung cancer. Clin Lung Cancer (2020) 21(5):e435–e44. doi: 10.1016/j.cllc.2020.02.025 32576443

